# Interobserver variability in assessing anterior cruciate ligament anatomy: comparing gross and microanatomical observations

**DOI:** 10.1007/s00276-026-03826-w

**Published:** 2026-02-06

**Authors:** Elizabeth Reschenberg, Aidan A. Ruth

**Affiliations:** 1 Department of Surgery, Center for Anatomical Science and Education, Saint Louis University School of Medicine, Saint Louis, MO USA; 2 A.T. Still University Kirksville College of Osteopathic Medicine, Kirksville, MO USA

**Keywords:** Interobserver error, Anterior cruciate ligament, Knee anatomy, Human anatomical variation

## Abstract

**Purpose:**

Most commonly, the ACL is described as having two distinct anatomical bundles: anteromedial and posterolateral. These bundles have been observed in a gross anatomical setting, but not as closely observed histologically. Here, we sought to compare observations of ACL bundle number when observed anatomically versus histologically to determine if the double bundle ACL is a reliably observed structure.

**Methods:**

Nineteen knees were dissected to expose the intracapsular ligaments. Bundle number was assessed in three ways: Via gross anatomical observation, histological observation, and via the presence of a connective tissue septum when observed histologically. Inter-rater agreements were calculated for each of the three modes. Cronbach’s alpha was calculated to assess the consistency of double-bundle observations across all three modes of observation.

**Results:**

Prevalence of a double-bundle ACL under gross observation was 68.4%. Inter-rater agreement was fair (Fleiss’s kappa = 0.202). Under histological observation, prevalence of a double-bundle ACL was 47.4%, with only 26.4% demonstrating a connective tissue septum that could be said to divide the ligament.

**Conclusion:**

These results suggest that the human ACL may exhibit more anatomical variation than has been previously appreciated in human cadaveric studies. Inter-rater agreement was fair, indicating that the demarcation of ACL bundles is not an obvious feature, even under gross observation. Additionally, our histological observations support the idea that ACL bundles exhibit substantial variation in terms of degree of distinct separation. This is, to our knowledge, the first study that has directly compared gross and microscopic observations of ACL bundle number.

## Introduction

The anterior cruciate ligament (ACL) is one of the most critical stabilizers of the knee joint, preventing excessive anterior translation of the tibia relative to the femur and guiding the tibia along its path of external rotation during gait [3, 8]. While the ACL has been recognized as having two bundles for nearly 200 years [21], there nevertheless remains unresolved questions about its anatomy. The two traditionally described bundles comprise the two functionally distinct components: the anteromedial (AM) and posterolateral (PL) bundles, named for their relative tibial insertion sites. These two bundles operate synergistically to stabilize the knee at different degrees of flexion and extension, with the AM bundle taut during flexion and the PL taut during extension and rotational movements [26, 27].

While the double bundle concept of the ACL is generally accepted, discrepancies exist in its observation. Gross anatomical studies vary in their ability to consistently demonstrate two distinct bundles, with variations attributable to differences in dissection technique or individual variation. In addition to the AM and PL bundles traditionally described, a third “intermediate” (IM) bundle was also first described by Norwood and Cross [15] as an additional support during rotational movements. Amis and Dawkins [3] confirmed the gross anatomical appearance of three bundles, but also noted that in younger specimens these bundles were difficult to separate and may have been the result of “wrinkling” upon flexion of the ligament in some instances. In other specimens, however, they noted that the three putative bundles were “demonstrably separate.” Otsubo et al. [17] described distinct attachments of these three bundles, while Kato et al. [10] explored these bundles biomechanically and found the IM bundle to act in support of the AM and PL bundles during rotation. Three bundles have been confirmed in at least a portion of the population radiologically [11]. Others have maintained that the ACL has either no discernable subdivisions at all [16, 22], or as many as 6–10 large “assemblies of fascicles” that cannot be grouped into distinct bundles [4, 13]. Ferretti’s study of the fetal development of the anterior cruciate ligament supports a double-bundle concept of the ACL, and is the first mention of a vascular connective tissue “septum” separating the two bundles histologically [7].

Histological studies of the anterior cruciate ligament have primarily focused on their division and landmarks near the femoral and tibial attachment sites rather than addressing bundle number explicitly [e.g., 5, 6, 28]. Strochi et al. [23] more generally described the ACL histologically and ultrastructurally as a collection of multiaxial collagen fibers ensheathed within connective tissue processes that divided the fibers into fascicles. While they described collagen and elastic fibers of different diameters and compositions, they were not correlated to location within the ligament itself. However, Suzuki and colleagues found differences in fiber dimensions in all three bundles, with the AM bundle primarily including thicker fibers oriented in one direction, the PL bundle including thinner fibers oriented in many different directions, and the intermediate bundle showing a transitional structure [25]. Recent work combining micro-CT and histological analysis has further highlighted the complex three-dimensional organization of the human ACL and the challenges inherent in defining discrete bundles across imaging and histological modalities [18]. These findings reinforce the need to critically evaluate how anatomical subdivisions are identified and interpreted across observational techniques.

Here, we take a novel approach to investigating the discrepancies between observed bundle numbers reported in the literature. Rather than investigating the variation within the ligament itself, here we investigate inter-observer variability when assessing the two bundles of the ACL. Inter-observer variability refers to differences or inconsistencies in observations made by different observers evaluating the same phenomenon. This variability can occur due to differences in training, experience, interpretation of criteria, or subjective judgment. Specifically, we investigate (1) variation in reported bundle number when observed grossly and (2) variation in reported bundle number when observed microscopically. Additionally, we investigate the presence or absence of a connective tissue septum when viewed microscopically.

## Materials and methods

### Study population

Nineteen knees were collected from the gross anatomy laboratory following the conclusion of an introductory gross anatomy course. Specimens were obtained through the Saint Louis University Gift Body Program. All donors provided informed consent for the use of their bodies in research and education in accordance with institutional and national guidelines. Because this study involved donated cadaveric material and did not constitute human subjects research, institutional review board approval was not required.

During this course, the joint capsule was opened and ligamentous anatomy observed, but no further dissection was performed. Knees from twelve females (mean age = 88.07 ± 10.76) and seven males (mean age = 89.06 ± 4.22) were included. Donors ranged in age from 74 to 109 at the time of death. Sample adequacy was assessed through effect size estimation and consistency metrics rather than retrospective power analysis.

### Observer selection and training

Gross anatomical observations were performed by six raters (two anatomy faculty and four graduate students), all of whom had prior formal training in knee anatomy but were not provided with specialized instruction regarding competing models of ACL bundle anatomy. Histological observations were performed by three graduate student raters who had completed formal coursework in histology and had experience as anatomy laboratory teaching assistants.

Before data collection, all raters were shown representative exemplars illustrating commonly described single- and double-bundle ACL configurations. Raters were instructed to independently evaluate each specimen and classify the ACL as either demonstrating two bundles (+) or not (−). No discussion among raters was permitted during scoring.

For gross anatomical assessment, raters evaluated specimens in person and were permitted to manipulate the knee freely, including flexion, extension, and rotation, to assist in bundle identification. For histological assessment, raters evaluated stitched composite micrographs representing full transverse sections of the ACL midsubstance.

### Gross anatomical preparation and observation

Prior to collection, the quadriceps femoris tendon and patellar retinacula had been reflected inferiorly to expose the joint cavity anteriorly. In preparation for observation, extraneous synovial tissue was removed (e.g., remnants of synovial plicae). Care was taken to avoid additional disruption of intra-articular structures.

Prior to the anatomy course, cadavers were embalmed via an arterial perfusion method using a proprietary embalming fluid of isopropyl alcohol (53%), Glycol (24%), Phenol (17%), and Formaldehyde (6%). After initial course use and following gross anatomical data collection, each ACL was marked with indelible ink to maintain orientation during histological processing and extracted from the knee. In addition to initial fixation prior to the dissection course, each specimen was fixed in 10% neutral buffered formalin for at least 48 h. Ligaments were removed from knees and trimmed to preserve the midsubstance of the ligament. These samples were then infiltrated and embedded with paraffin wax. Transverse sections were cut at 7 μm with a rotary microtome, floated in a water bath, and mounted on positively charged slides. Individual slides were stained using picrosirius red according to manufacturer’s protocol (PolySciences, Inc.). In brightfield microscopy, collagen fibers stain red with picrosirius red.

### Light microscopy

A Leica DMi1 light microscope was used to visualize the histological sections of each specimen. Representative sections for each of the 19 specimens were photographed to be used in later observations. Using the LAS X software associated with the microscope, micrographs from each of the representative sections were captured and stitched to create a composite of the entire section.

### Statistical analysis

Inter-relater reliability, or the degree of agreement between observers, was determined for each separate test of bundle number (i.e., gross anatomical, histological observation, and presence or absence of septum) using Fleiss’s kappa [12]. Reliable raters are said to agree with one another and were interpreted using commonly accepted benchmarks for agreement [20].

“Prevalence” of a double bundle was calculated for each of the three modes of observation. This statistic was calculated as the percentage of the total sample for which at least two of three raters agreed on the presence of a double bundle.

Cronbach’s alpha was calculated to assess the consistency of double bundle observations across all three modes of observation: gross anatomical, histological, and presence or absence of a septum. Cronbach’s alpha is typically used to assess the internal consistency of survey instruments by comparing the covariance among items comprising the survey instrument to the amount of overall variance. Here, we compared the covariance among each of three modes of observation (gross anatomical, histological, and presence or absence of a septum) with the overall variance. Calculations were performed using SPSS statistics v. 28 (IBM).

## Results

**Fig. 1:**
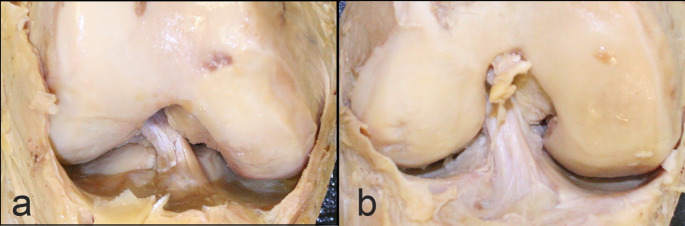
All observers agreed that specimen 23015 presented a single bundle (**a**). All observers likewise agreed that specimen 23065 presented a double bundle (**b**). Note that the knees photographed here are in different anatomical positions, but that observations of bundle were made from dynamic handling and observing specimens in person and not from photographs

Summary statistics for each mode of observation are presented in Table 1. Thirteen out of nineteen specimens (68.4%) demonstrated a double-bundle ACL according to our raters. Figure 1 presents specimens for which all observers agreed on either the presence of a single bundle (Fig. 1A) or a double bundle (Fig. 1B). Inter-observer agreement as measured by Fleiss’s kappa was found to be 0.198 (*p* = 0.135), indicating poor agreement.

**Table 1:** Summary statistics for different modes of observation of the ACL

Mode of observation	Prevalence (%)	Kappa	Confidence interval	Significance
Gross Anatomy	68.4	0.198 (Fair agreement)	−0.061 to 0.458	0.135
Histology	47.4	0.438 (Moderate agreement)	0.179 to 0.698	< 0.001
Histology – Septum	26.3	0.684 (Substantial agreement)	0.425 to 0.944	< 0.001

The prevalence of a double bundle when observed with histology was 47.4% (9 out of 19). Fleiss’s kappa was 0.438 (*p* < 0.001) indicating moderate agreement between observers. The prevalence of a vascular connective tissue septum dividing the ACL into different bundles was 26.3% (5/19). Fleiss’ kappa for the presence of a septum was 0.684 (*p* = 0.001), indicating substantial agreement between observers. Pairwise McNemar tests revealed no statistically significant differences between faculty raters and student raters (all *p* > 0.05), indicating that interobserver variability was not driven by systematic differences in individual rater judgments.

**Fig. 2:**
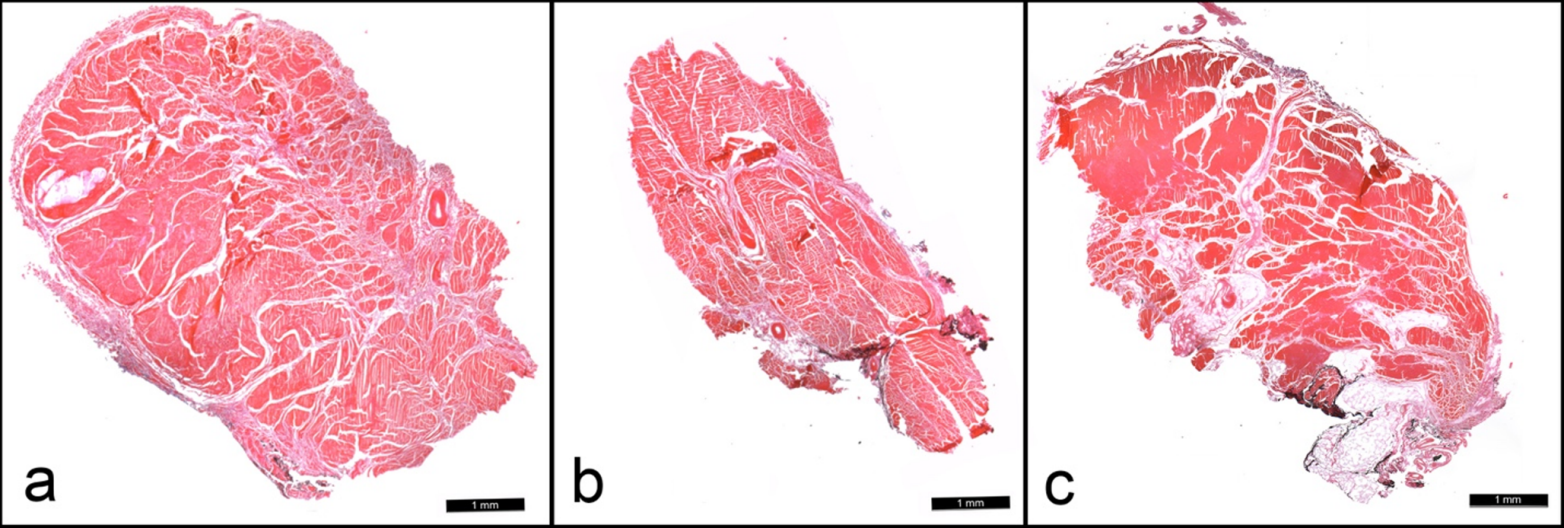
**a** All observers agreed that Specimen 22327 presented a single bundle without a connective tissue septum. **b** All observers agreed that Specimen 22364 presented a double bundle without a connective tissue septum. **c** All observers agreed that Specimen 22337 presented a double bundle with connective tissue septum.

Figure 2 presents specimens for which all observers agreed on the presence of a single bundle (Fig. 2a), a double bundle without a connective tissue septum (Fig. 2b), and a double bundle with a connective tissue septum (Fig. 2c).

## Comparison of observational modes

When gross anatomical observation, histological observation, and histological septum identification were considered together, consistency across observational modalities was acceptably reliable (α = 0.67). This indicates that specimens identified as having a double bundle by one method were more likely to be similarly classified using the other methods, despite variability in absolute prevalence and agreement levels.

## Discussion

Our results showed that 68.4% of knees exhibited a double-bundle ACL under gross observation, but agreement among observers was poor. Even among anatomically trained observers, the identification of discrete bundles was inconsistent, suggesting that bundle distinction is not always a grossly apparent feature. In contrast, histological examination yielded fewer observations of double bundles (47.4%), but with higher inter-rater agreement. Only 26.3% of specimens demonstrated a connective tissue septum histologically, suggesting that while gross anatomical impressions might favor a double-bundle model, histological validation remains limited. We also found that our three modes of observation were acceptably reliable when covariance was compared with overall variance. This means that an ACL that was observed to have a double bundle in one mode of observation was more likely to also have double bundles in the other modes of observation.

The current study has some limitations. Our cohort of observers included students, who, although advanced, may not have appreciated the anatomy of the double bundles adequately. Additionally, since our knees had previously been dissected in a traditional dissection course, some damage may have occurred to the ligaments prior to the study. However, we did not observe any such damage. Although prolonged fixation prior to histological preparation is not an ideal protocol for many histological methods, many studies have shown that cadavers embalmed for teaching can also be used for routine histology such as the protocols used in this study [1, 14]. Because we only explored the midsubstance of the ligament and not further distally or proximally, we may have captured the area of the ligament with the least distinction between the AM and PL bundles, thus falsely diminishing the prevalence.

Several factors may contribute to variable observation of ACL bundles. First, the relatively advanced age of our cadaveric sample (mean age ~ 88 years) may have affected ACL morphology. Age-related remodeling, degeneration, or coalescence of fascicular structures could obscure distinctions between bundles. Prior studies have shown that connective tissues undergo structural changes with age, which may include diminished vascularity and collagen reorganization [7].

Sex-based differences in ACL structure also warrant consideration. Although the present study did not seek to address sex differences specifically, existing literature demonstrates that females experience higher rates of ACL injury [2]. While some literature suggests that this may be due to ligament composition [9], alternative explanations include differences in gross anatomical features such as quadriceps angle and tibial plateau [24] and differences in strength training and conditioning between male and female athletes [19]. Whether such differences translate into distinct gross or histological bundle morphology remains incompletely understood and represents and important avenue for future investigation.

The observations that we report here support Ferretti and colleagues’ report of a vascular connective tissue septum in the fetal ACL [7]. This septum persists in at least some of our sample of aged adults. However, its absence in the majority of our sample suggests that it may be a structure that is remodeled throughout life in at least some individuals. Ferretti and colleagues argue that the orientation of a histological sample may not always be appropriate to observe the septum or the two bundles. However, our samples were prepared in the suggested transverse orientation, and still did not always demonstrate these structures. Our observations may suggest that the human ACL initially begins as two distinct ligamentous structures that remodel and coalesce into one another throughout life. What may cause this remodeling, whether activity patterns, microinjury and healing, allometric constraints, or some other force, is unknown.

Another possibility is that, while the bundles are not always anatomically separate, they nevertheless perform distinct biomechanical functions depending on orientation of individual collagen fascicles or groups of fascicles. This interpretation is compatible with the double bundle concept of the ACL in that the majority of these fibers may be oriented in two distinct orientations but still accommodates the “assemblies of fascicles” reported by Mommersteeg [13] as well as the occasional presence of the intermediate bundle. The findings of Parrilli et al., who demonstrated substantial microstructural heterogeneity of the ACL using combined micro-CT and histology [18], align with our observations of variable bundle identification and limited histological demarcation. Together, these studies suggest that discrepancies in reported ACL bundle anatomy may reflect both true biological variation and methodological constraints.

The clinical implications of these findings lie in the assumption that a double-bundle ACL is a standard anatomical norm, which has informed reconstruction techniques. Our findings challenge this notion, suggesting that anatomical variation and subjective interpretation may play a larger role than previously appreciated. Future surgical planning may benefit from individualized assessment using imaging or intraoperative observation rather than assuming a uniform double-bundle configuration. Additionally, our results may call for a reassessment of anatomical models used in both surgical education and biomechanical studies.

In the realm of research practice, we hope to underscore the anatomical complexity and variability of the ligament, and that our results encourage researchers to collect rigorous observational data. Moreover, the observed presence of a vascular connective tissue septum in only 26% of specimens, despite evidence of it in fetal ACLs, may suggest that the ligament undergoes significant remodeling throughout life. Areas for further investigation may include exploring how these changes impact ACL function, injury risk, and recovery, particularly across different age groups. Our findings also underscore the need for more refined imaging and diagnostic tools to accurately assess individual ACL anatomy, potentially leading to more personalized surgical and rehabilitation approaches.

## Data Availability

No datasets were generated or analysed during the current study.

## References

[1] Abuhaimed AK, Almulhim AM, Alarfaj FA et al (2020) Histologic reliability of tissues from embalmed cadavers: Can they be useful in medical education? Saudi J Med Med Sci 8:208–212 32952513 10.4103/sjmms.sjmms_383_19 PMC7485655

[2] Agel J, Rockwood T, Klossner D (2016) Collegiate ACL injury rates across 15 sports: National collegiate athletic association injury surveillance system data update (2004–2005 through 2012–2013). Clin J Sports Med 26:518–523. 10.1097/JSM.0000000000000290 27315457

[3] Amis AA, Dawkins GP (1991) Functional anatomy of the anterior cruciate ligament. J Bone Jt Surg 73–B:1–8 10.1302/0301-620X.73B2.2005151 2005151

[4] Arnoczky SP (1983) Anatomy of the anterior cruciate ligament. Clin Orthop Relat Res 173:19–25 6821989

[5] Beaulieu ML, Carey GE, Schlecht SH, Wojtys EM, Ashton-Miller JA (2015) Quantitative comparison of the microscopic anatomy of the human ACL femoral and tibial entheses. J Orthop Res 33:1811–1817 26134706 10.1002/jor.22966 PMC4628572

[6] Beaulieu ML, Carey GE, Schlecht SH, Wojtys EM, Ashton-Miller JA (2016) On the heterogeneity of the femoral enthesis of the human ACL: microscopic anatomy and clinical implications. J Exp Orthop 3:1–9 10.1186/s40634-016-0050-8 PMC4943914 27412665

[7] Ferretti M, Levicoff EA, Macpherson TA, Moreland MS, Cohen M, Fu FH (2007) The fetal anterior cruciate ligament: an anatomic and histologic study. Arthroscopy 23:278–283 17349471 10.1016/j.arthro.2006.11.006

[8] Flandry F, Hommel G (2011) Normal anatomy and biomechanics of the knee. Sports Med Arthrosc Rev 19:82–92 21540705 10.1097/JSA.0b013e318210c0aa

[9] Hashemi J, Mansouri H, Chandrashekar N et al (2011) Age, Sex, body Anthropometry, and ACL size predict the structural properties of the human anterior cruciate ligament. J Orthop Res 29:993–1001 21246609 10.1002/jor.21245

[10] Kato Y, Ingham SJM, Maeyama A, Lertwanich P, Wang JH, Mifune Y et al (2012) Biomechanics of the human triple-bundle anterior cruciate ligament. Arthroscopy 28:247–254 22019233 10.1016/j.arthro.2011.07.019

[11] MacKay JW, Whitehead H, Toms AP (2014) Radiological evidence for the triple bundle anterior cruciate ligament. Clin Anat 27:1097–1102 24890455 10.1002/ca.22420

[12] McKenzie KA, Mahnken JD (2024) Estimator of agreement with covariate adjustment. J Agric Biol Environ Stat 29:19–35

[13] Mommersteeg TJ, Kooloos JG, Blankevoort L, Kauer JM, Huiskes R, Roeling FQ (1995) The fibre bundle anatomy of human cruciate ligaments. J Anat 187:461–471 7592008 PMC1167440

[14] Nicholson HD, Samalia L, Gould M, Hurst PR, Woodroffe M (2005) A comparison of different embalming fluids on the quality of histological preservation in human cadavers. Eur J Morphol 42:178–184 16982474 10.1080/09243860500473306

[15] Norwood LA, Cross MJ (1979) Anterior cruciate ligament: functional anatomy of its bundles in rotatory instabilities. Am J Sports Med 7:23–26 420384 10.1177/036354657900700106

[16] Odensten M, Gillquist J (1985) Functional anatomy of the anterior cruciate ligament and a rationale for reconstruction. J Bone Joint Surg Am 67:257–262 3968118

[17] Otsubo H, Shino K, Suzuki D, Kamiya T, Suzuki T, Watanabe K et al (2011) The arrangement and the attachment areas of three ACL bundles. Knee Surg Sports Traumatol Arthrosc 20:127–134 21695467 10.1007/s00167-011-1576-z

[18] Parrilli A, Grassi A, Orellana F et al (2024) 3D visualization of the human anterior cruciate ligament combining micro-CT and histological analysis. Surg Radiol Anat 46:249–258 38265490 10.1007/s00276-023-03295-5 PMC10861685

[19] Parsons JL, Coen SE, Bekker S (2021) Anterior cruciate ligament injury: towards a gendered environmental approach. Br J Sports Med 55:984–990 33692033 10.1136/bjsports-2020-103173

[20] Saal FE, Downey RG, Lahey MA (1980) Rating the ratings: assessing the psychometric quality of rating data. Psychol Bull 88:413–428

[21] Schindler OS (2012) The story of anterior cruciate ligament reconstruction – Part 1. J Perioper Pract 22:163–171 22720509 10.1177/175045891202200505

[22] Smigielski R, Zdanowicz U, Drwięga M, Ciszek B, Ciszkowska-Łysoń B, Siebold R (2014) Ribbon-like appearance of the midsubstance fibres of the anterior cruciate ligament close to its femoral insertion site: a cadaveric study including 111 knees. Knee Surg Sports Traumatol Arthrosc 23:3143–3150 24972997 10.1007/s00167-014-3146-7 PMC4611008

[23] Strocchi R, De Pasquale V, Gubellini P, Facchini A, Marcacci M, Buda R et al (1992) The human anterior cruciate ligament: histological and ultrastructural observations. J Anat 180:515–519 1487443 PMC1259652

[24] Sutton KM, Bullock JM (2013) Anterior cruciate ligament rupture: differences between males and females. J Am Acad Orthop Surg 21:41–50 23281470 10.5435/JAAOS-21-01-41

[25] Suzuki D, Otsubo H, Watanabe T, Kamiya T, Nagoya S, Yamashita T et al (2015) Ultrastructure of the three anterior cruciate ligament bundles. Clin Anat 28:910–916 26118465 10.1002/ca.22586

[26] Takahashi M, Doi M, Abe M, Suzuki D, Nagano A (2017) Anatomical study of the femoral and tibial insertions of the anteromedial and posterolateral bundles of the human anterior cruciate ligament. Am J Sports Med 34:787–792 10.1177/0363546505282625 16452272

[27] Zantop T, Herbort M, Raschke MJ, Fu FH, Petersen W (2017) The role of the anteromedial and posterolateral bundles of the anterior cruciate ligament in anterior tibial translation and internal rotation. Am J Sports Med 35:223–227 10.1177/0363546506294571 17158275

[28] Zauleck MK, Gabriel S, Fischmeister MF, Hirtler L (2014) Origin of the anterior cruciate ligament and the surrounding osseous landmarks of the femur. Clin Anat 27:1103–1110 25065356 10.1002/ca.22440

